# Amino acid substitutions involved in the adaptation of a novel highly pathogenic H5N2 avian influenza virus in mice

**DOI:** 10.1186/s12985-016-0612-5

**Published:** 2016-09-23

**Authors:** Haibo Wu, Xiuming Peng, Xiaorong Peng, Nanping Wu

**Affiliations:** State Key Laboratory for Diagnosis and Treatment of Infectious Diseases, Collaborative Innovation Center for Diagnosis and Treatment of Infectious Diseases, the First Affiliated Hospital, School of Medicine, Zhejiang University, 79 Qingchun Road, 310003 Hangzhou, Zhejiang China

**Keywords:** Avian influenza viruses, H5N2, Mice, Adaptation, Virulence, Replication

## Abstract

**Background:**

H5N2 avian influenza viruses (AIVs) can infect individuals that are in frequent contact with infected birds. In 2013, we isolated a novel reassortant highly pathogenic H5N2 AIV strain [A/duck/Zhejiang/6DK19/2013(H5N2) (6DK19)] from a duck in Eastern China. This study was undertaken to understand the adaptive processes that led enhanced replication and increased virulence of 6DK19 in mammals. 6DK19 was adapted to mice using serial lung-to-lung passages (10 passages total). The virulence of the wild-type virus (WT-6DK19) and mouse-adapted virus (MA-6DK19) was determined in mice. The whole-genome sequences of MA-6DK19 and WT-6DK19 were compared to determine amino acid differences.

**Findings:**

Amino acid changes were identified in the MA-DK19 PB2 (E627K), PB1 (I181T), HA (A150S), NS1 (seven amino acid extension “WRNKVAD” at the C-terminal), and NS2 (E69G) proteins. Survival and histology analyses demonstrated that MA-6DK19 was more virulent in mice than WT-6DK19.

**Conclusion:**

Our results suggest that these substitutions are involved in the enhanced replication efficiency and virulence of H5N2 AIVs in mammals. Continuing surveillance for H5N2 viruses in poultry that are carrying these mutations is required.

**Electronic supplementary material:**

The online version of this article (doi:10.1186/s12985-016-0612-5) contains supplementary material, which is available to authorized users.

## Findings

Highly pathogenic H5 avian influenza viruses (AIVs) emerged from Asia in 2003 and have caused severe epidemics among poultry and humans [1–4]. Of the 850 human cases reported to the World Health Organization as of April 4, 2016, 449 (52.8 %) were fatal [5]. Given that highly pathogenic H5 AIVs continue to cross into the human population and that humans lack pre-existing immunity to the viruses, there is the possibility that a pandemic human influenza virus will emerge.

Live poultry markets (LPMs), sites for the sale and slaughter of domestic poultry in East Asia [6, 7], are major venues for AIV dissemination, influenza virus reassortment, and cross-species transfer of AIVs [6, 8–10]. H5N2 AIVs are consistently found in poultry from LPMs [4, 11, 12] and transmission to individuals in frequent contact with infected birds has been well documented [13, 14]. In addition to active surveillance of LPMs for emergent AIVs, it is necessary to understand the adaptive processes that cause H5N2 AIVs to become highly pathogenic (defined as enhanced replication and increased virulence) in mammals.

Our laboratory has previously isolated a novel reassortant highly pathogenic H5N2 AIV [A/duck/Zhejiang/6DK19/2013 (H5N2) (6DK19)] from an apparently healthy domestic duck from a LPM [11]. This study was undertaken to investigate the amino acid substitutions associated with adaptation of 6DK19 to mammals, and to determine the virulence of mouse-adapted 6DK19 in vivo.

All of the animal experiments described in this study were approved by the Ethics Committee of the First Affiliated Hospital, School of Medicine, Zhejiang University (No. 2015-15). 6DK19 was adapted to a murine host by serial lung-to-lung passage (10 passages) of the wildtype (WT) 6DK19 virus as described previously [15, 16] to obtain the mouse-adapted virus [A/duck/Zhejiang/6DK19-mouse-adapted/2013(H5N2), MA-6DK19]. Six-week-old female BALB/c mice (*n* = 5) were inoculated intranasally with 10^6.0^ EID50 (50 % embryo infectious dose) of 6DK19 in 50 μL of phosphate buffered saline (PBS). Based on previously published studies, 6DK19 was moderately pathogenic in mice [11]. Mice were sacrificed at 3 days post-inoculation (dpi) and the lungs were harvested in 1 mL of PBS. The lung tissue was disrupted and then centrifuged. Fifty microliters (50 μL) of supernatant was used to inoculate the subsequent naïve mouse in the series. The pathogenicities of WT-DK619 and MA-DK619 were tested in 15 6-week-old female BALB/c mice inoculated intranasally with 10^6.0^ EID50 (50 μL). Three mice were sacrificed from each group at 3, 6, and 9 dpi, and the viral titer in the lung, brain, heart, liver, kidney, and spleen was determined in embryonated chicken eggs by the Reed and Muench method [17]. Survival and weight-loss were monitored in the remaining six mice in each group until 14 dpi. A group of mock-infected mice (*n* = 6) was included as a control. All experiments with the H5N2 viruses were performed in a Biosafety Level 3 laboratory.

Lung tissue samples from WT-6DK19 or MA-6DK19 infected mice were fixed in 10 % neutral buffered formalin, embedded in paraffin, then cut into 4 μm-thick sections and stained with hematoxylin and eosin (H&E). Immunohistochemical staining was performed to detect nucleoprotein antigens in the lungs. The tissues were incubated overnight at 4 °C with a monoclonal antibody against the influenza A virus nucleoprotein, then the sections were washed 3 times with PBS and incubated with an HRP–conjugated goat anti–mouse secondary antibody. The sections were developed with 3–3′ diaminobenzidine and examined under a light microscope as described previously [18].

To identify the virulence-associated molecular markers of MA-6DK19, the whole genomes of MA-6DK19 and WT-6DK19 were sequenced and compared to identify amino acid changes. Viral RNA was extracted from the supernatant of the disrupted lung tissue using TRIzol. The Uni12 primer was used to synthesize cDNA from viral RNA: 5ʹ-AGCAAAAGCAGG-3ʹ. RT-PCR was conducted using a PrimeScript™ 1st Strand cDNA Synthesis Kit and PrimeSTAR® Max DNA Polymerase (TaKaRa). All of the gene segments from WT-6DK19 and MA-6DK19 were amplified with segment-specific primers as described previously [19]. All eight segments of these viruses sequenced using Sanger sequencing on an ABI 3730 genetic analyser (Applied Biosystems). The sequences were analysed using BioEdit version 7.0.9.0 DNA software. The sequence data of WT-6DK19 and MA-6DK19 have been deposited in GenBank (accession nos. KJ933374-KJ933381 and KX714303-KX714310).

**Fig. 1:**
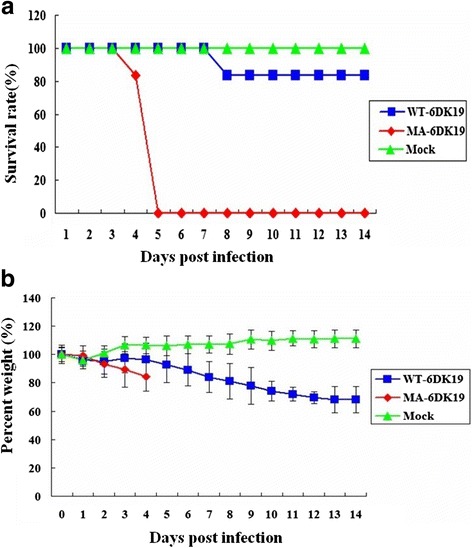
Survival and body weight were measured in mice infected with the H5N2 viruses. Survival (**a**) and body weight (**b**) were measured in BABL/c mice infected with the wild-type (WT-6DK19) or mouse-adapted (MA-6DK19) strains of an H5N2 avian influenza virus (*n* = 6/group). Each mouse was infected intranasally with 10^6.0^ EID_50_ of virus in a 50 μL volume. The number of surviving mice and their body weights were measured daily from the date of challenge to 14 days post inoculation

**Fig. 2:**
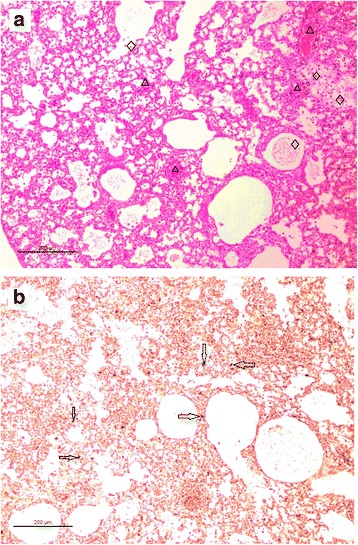
Histology and immunohistochemistry of mice infected with the mouse-adapted H5N2 avian influenza virus. Lung pathology was determined in mice infected with mouse-adapted strain of an H5N2 avian influenza virus at 3 days post inoculation (dpi) (**a**). Hematoxylin and eosin staining was used to examine the histology of the lung tissue. Mice infected with the mouse-adapted virus displayed severe interstitial pneumonia in lung tissues, shown by the alveolar lumen flooded with dropout from alveolar cells, erythrocytes, and inflammatory cells (diamond); and congestion in the blood vessels (triangle). Viral nucleoprotein was detected in the lungs using immunohistochemistry in mice infected with the mouse-adapted viruses (**b**). Arrows indicate positively stained lung alveolar epithelial cells

Amino acid substitutions that increase the virulence of H5 AIVs adapted to mammalian hosts have been shown to emerge after the fifth or sixth passage through naïve mice [20, 21]. Here, some of these mutations were detected as early as the fourth passage (Table 1 and Additional file 1: Figure S1). In contrast to mice infected with WT-MDK19 that exhibited minimal weight loss, mice infected with MA-6DK19 exhibited rapid weight-loss beginning on 2 dpi (Fig. 1) and had clear clinical signs of illness. The survival rate for mice infected with WT-6DK19 was 83.3 % (5/6) up to 14 dpi (Table 2). In contrast, none of mice infected with MA-6DK19 survived to 14 dpi, indicating that MA-6DK19 is more virulent in mice than WT-6DK19. Mice infected with MA-6DK19 had multifocal severe interstitial inflammatory hyperaemia and exudative pathological changes, large lesions in the lung tissue, and red blood cell and inflammatory cell infiltrates at 3 dpi (Fig. 2). Cells infected with H5N2 AIV were detected in the bronchial epithelium and alveolar epithelium from infected mice 3 dpi.

**Table 1:** Nucleotide and amino acid substitutions identified in a mouse-adapted highly pathogenic H5N2 avian influenza virus

Segment	Nucleotide position	Nucleotide substitution		Amino acid position	Amino acid substitution
PB2	1879 (G → A)	Passage 2 (P2)	G	627(E → K)	E
		P3	G/A		E/K
		P4	A		K
		P10	A		K
PB1	542 (T → C)	P7	T	181 (I → T)	I
		P8	C		T
		P10	C		T
	2103	T → C		701	-
	2112	A → G		704	-
HA	448 (G → T)	P6	G	150 (A → S)	A
		P7	G/T		A/S
		P8	T		S
		P10	T		S
NS	678 (A → G)	P8	A	NS1, 226 (Terminator → W)	Terminator
				NS2, 69 (E → G)	E
		P9	A/G	NS1, 226	Terminator/(W, “WRNKVAD” was extended)
				NS2, 69	E/G
		P10	G	NS1, 226	W, “WRNKVAD” was extended
				NS2, 69	G

“-” Synonymous substitution

**Table 2:** Viral titer of wild-type and mouse-adapted H5N2 avian influenza viruses in tissue

Virus	Days post-infection	Virus titers in organs of mice (log10 EID_50_/mL) no. virus-positive mice/no. tested mice (mean titer ± SD)					
		Lung	Spleen	Kidney	Liver	Brain	Heart
Wild-type virus (WT-6DK19)	3	3/3(4.0 ± 0)	1/3(2.0 ± 0)	1/3(2.0 ± 0)	3/3(1.5 ± 0.5)	2/3(2.5 ± 0.5)	3/3(1.5 ± 0.5)
	6	3/3(4.5 ± 0.5)	2/3(2.0 ± 0.0)	3/3(2.0 ± 0)	2/3(2.0 ± 0)	3/3(1.5 ± 0.5)	3/3(2.0 ± 0)
	9	3/3(3.5 ± 0)	2/3(2.0 ± 0.0)	0/3	2/3(1.0 ± 0)	3/3(1.5 ± 0.5)	3/3(2.0 ± 0)
Mouse-adapted virus (MA-6DK19)	3	3/3(5.5 ± 0.5)	3/3(2.5 ± 0.5)	3/3(3.0 ± 0)	3/3(2.5 ± 0.5)	3/3(2.5 ± 0.5)	3/3(3.0 ± 0)
	6	ND	ND	ND	ND	ND	ND
	9	ND	ND	ND	ND	ND	ND

Fifteen (15) mice/group were inoculated intranasally with 10^6.0^ EID_50_ of either the wild-type (WT-6DK19) or mouse-adapted (MA-6DK19) viruses in a 50 μL volume. Three mice per group were sacrificed at 3, 6, or 9 dpi and the lung, brain, heart, kidney, spleen, and liver tissues were collected. The viral titer in each tissue was determined in embryonated chicken eggs by the Reed and Muench method. Values represent mean ± SD. ND: Not determined. None of mice infected with MA-6DK19 survived past 5 dpi

During the adaptation process, six nucleotide substitutions and five amino acid substitutions were observed (Table 1): (1) an E → K substitution in polymerase basic protein 2 (PB2) at position 627, (2) an I → T substitution in polymerase basic protein 1 (PB1) at position 181, (3) an A → S substitution in hemagglutinin (HA) at position 150, (4) seven amino acids (WRNKVAD) were added at the C-terminal of the nonstructural protein 1 (NS1), and (5) an E → G substitution in NS2 at position 69. The E627K substitution in the PB2 protein has been reported to influence host range and to confer increased virulence in models of H3, H5, H6, and H9 infection [22–25]. The A149 (or 150) substitution has been reported to be involved in the 150-loop of the receptor binding domain and is implicated in the adaptation of AIVs to mammalian hosts [26, 27]. Previously, the C-terminal ESEV motif has been shown to increase viral virulence when introduced into the NS1 protein of mouse-adapted influenza virus in a strain dependent manner [28, 29]. The significance of the seven amino acid addition to MA-6DK19 NS1 is not entirely clear [30], and it has been observed frequently in H5N8 viruses in recent years (Additional file 2: Table S1). Compared to WT-6DK19, the mouse-adapted virus had expanded tissue tropism and increased replication kinetics in vivo; however, the substitutions that contributed to mouse adaptation remain to be further studied.

Mice are widely used to study the pathogenesis of AIVs [25, 31]. Several amino acid substitutions including PB2 (Q591K and D701N), polymerase acidic protein (PA) (I554V), HA (S227N), and NP (R351K) have been described in mouse adapted H5N2 AIVs that have increased virulence and enhanced replication kinetics in mice and cell lines [20]. In this study, amino acid substitutions, in the PB2 (E627K), PB1 (I181T), HA (A150S), NS1 (WRNKVAD was extended at the C-terminal of the protein), and NS2 (E69G) proteins were identified in a MA-6DK19. These changes were associated with increased virulence compared with the wild-type virus, and the mouse-adapted virus became lethal in mice. These results provide insights into the pathogenic potential of novel reassortant H5N2 AIVs in mammals, and suggest that continued H5N2 molecular epidemiology studies are critical to understand the variability and evolutionary mechanisms of AIVs.

## Additional files

Additional file 1: Figure S1. Comparison of the PB2, PB1, HA, and NS segment sequences of the H5N2 viruses in differnet passages. (DOC 316 kb) Additional file 2: Table S1. Amino acid substitutions in PB2, PB1, HA, NS1 and NS2 proteins of H5 influenza A viruses. (DOC 76 kb)

## Abbreviations

AIVs: Avian influenza viruses
dpi: Days post-inoculation
EID50: 50 % embryo infectious dose
HA: Hemagglutinin
LPMs: Live poultry markets
MA: Mouse-adapted
PA: Polymerase acidic protein
PB1: Polymerase basic protein 1
PB2: Polymerase basic protein 2
PBS: Phosphate buffered saline
WT: Wildtype
