# Supplementary figures and images for: Amino acid substitutions involved in the adaptation of a novel highly pathogenic H5N2 avian influenza virus in mice
Source: Virol J. 2016 Sep 23;13:159. doi: 10.1186/s12985-016-0612-5 (PMC5035443; doi:10.1186/s12985-016-0612-5)

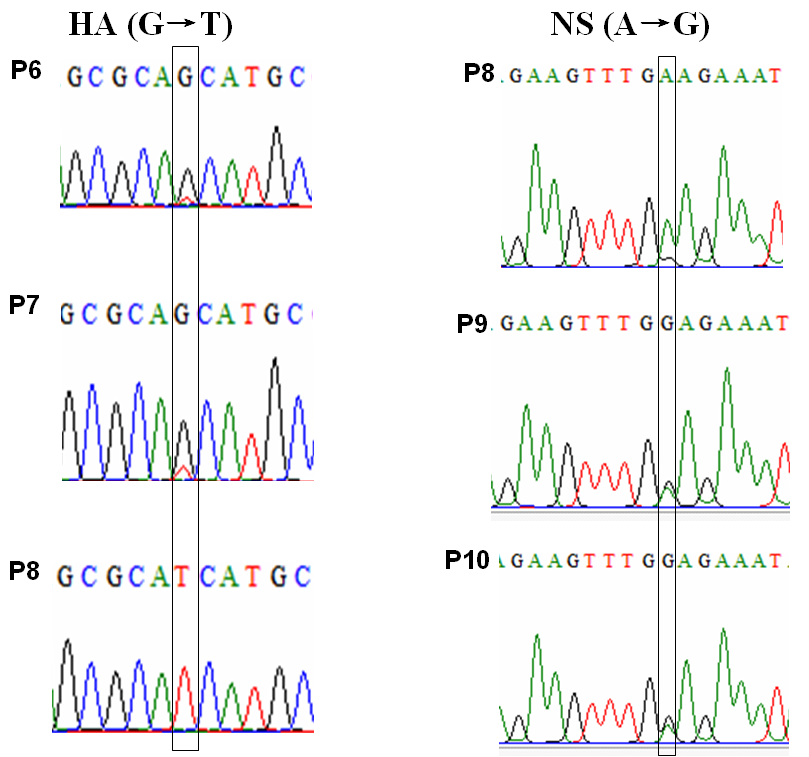


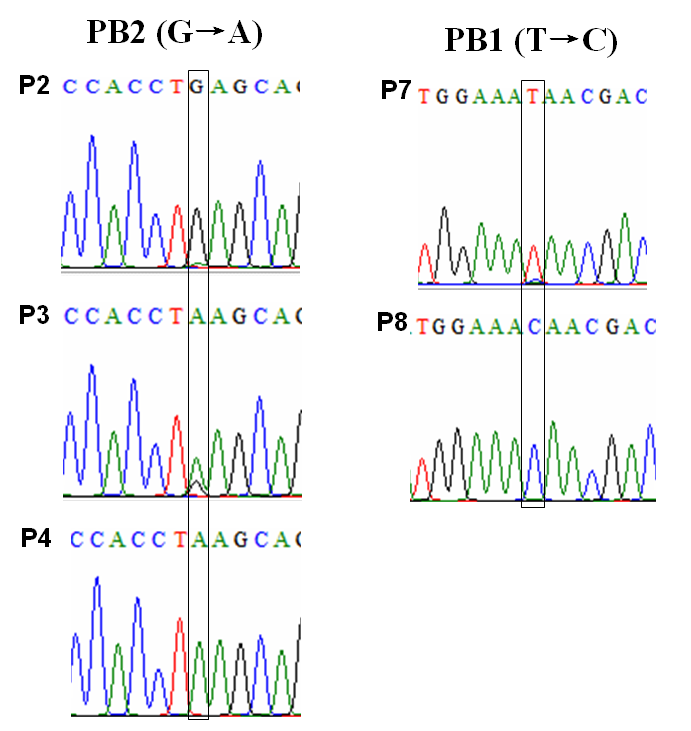

Supplement: Additional file 1: Figure S1. — Comparison of the PB2, PB1, HA, and NS segment sequences of the H5N2 viruses in differnet passages. (DOC 316 kb) [file 12985_2016_612_MOESM1_ESM.doc]
