# Supplementary material for: Amino acid substitutions involved in the adaptation of a novel highly pathogenic H5N2 avian influenza virus in mice
Source: Virol J. 2016 Sep 23;13:159. doi: 10.1186/s12985-016-0612-5 (PMC5035443; doi:10.1186/s12985-016-0612-5)
Supplement: Additional file 2: Table S1. — Amino acid substitutions in PB2, PB1, HA, NS1 and NS2 proteins of H5 influenza A viruses. (DOC 76 kb) [file 12985_2016_612_MOESM2_ESM.doc]

**Table S1 Amino acid substitutions in PB2, PB1, HA, NS1 and NS2 proteins of H5 influenza A viruses.**

|  | **H5N2** | **H5N1** | **H5N3** | **H5N4** | **H5N5** | **H5N6** | **H5N7** | **H5N8** | **H5N9** |
| --- | --- | --- | --- | --- | --- | --- | --- | --- | --- |
| **PB2-627E** | Avian (683), Others (24). Total, 707, 99.16%(707/713). | Avian (1911), Human(188), Tiger(7), Swine(27), Cat(1), Others (100).  Total, 2234, 79.02%(2234/2827). | Avian (96), Others (11).  Total,107,100% (107/107). | Avian (5), Others (1).  Total,6,100% (6/6). | Avian (40).  Total,40,100% (40/40). | Avian (92), Human(1), Others (23).  Total, 116, 98.31%(116/118). | Avian (10). Total,10,100% (10/10). | Avian (163), Others (24). Total,187,100% (187/187). | Avian (26). Total,26,100% (26/26). |
| **PB2-627K** | Avian (6). Total, 6, 0.84%(6/713). | Avian (491), Human(84), Tiger(6), Canine(1), Cat(2), Equine(1), Ferret(6), Mink(1), Stone marten(1).  Total, 593, 20.98%(593/2827). | 0 | 0 | 0 | Human(2),  Total, 2, 1.69%(2/118). | 0 | 0 | 0 |
|  |  |  |  |  |  |  |  |  |  |
| **PB1-181 I** | Avian (722), Others (25). Total,747,100% (747/7457). | Avian (2343), Human(271), Swine(25), Tiger(9),  Others (386).  Total, 2761, 98.40%(2761/2806). | Avian (96),Others (11).  Total, 107, 99.07%(107/108). | Avian (6).  Total, 6, 100%(6/6). | Avian (40).  Total, 40, 100%(40/40). | Avian (93), Human(3),  Others (24). Total, 120, 100%(120/120). | Avian (10). Total,10,100% (10/10). | Avian (163), Others (24). Total,187,100% (187/187). | Avian (26). Total,26,100% (26/26). |
| **PB1-181T** | 0 | 0 | 0 | 0 | 0 | 0 | 0 | 0 | 0 |
| PB1-181V | 0 | Avian (41).  Total, 41, 1.46%(41/2806) | Avian (1). Total, 1, 0.93%(1/108) | 0 | 0 | 0 | 0 | 0 | 0 |
| PB1-181M | 0 | Avian (3), Swine(1). Total, 4, 0.14%(4/2806) | 0 | 0 | 0 | 0 | 0 | 0 | 0 |
|  |  |  |  |  |  |  |  |  |  |
| **HA-150A** | Avian (932), Others (28). Total,960,100% (960/960). | Avian (5114), Human(445), Swine(34), Tiger(15), Others (203).  Total,5811,99.74%(5811/5826) | Avian (142), Others (11). Total,153,100% (153/153). | Avian (4), Others (1).  Total,5,100% (5/5). | Avian (42).  Total, 42, 100%(42/42). | Avian (144), Human(3).  Total, 147, 100%(147/147). | Avian (14). Total,14,100% (14/14). | Avian (192), Others (23). Total,215,100% (215/215). | Avian (33). Total,33,91.67% (33/36). |
| **HA-150S** | 0 | Human(1), Swine(1).  Total, 2, 0.04%(2/5826). | 0 | 0 | 0 | 0 | 0 | 0 | Avian (3). Total,3,8.33% (3/36). |
| HA-150V | 0 | Avian (1), Human(9).  Total, 10,0.17%(10/5826). | 0 | 0 | 0 | 0 | 0 | 0 | 0 |
| HA-150T | 0 | Avian (1), Human(2).  Total, 3, 0.05%(3/5826). | 0 | 0 | 0 | 0 | 0 | 0 | 0 |
|  |  |  |  |  |  |  |  |  |  |
| **NS1-226 Terminator** | Avian (688), Others (27).  Total,715,92.00% (715/777). | Avian (2373), Human(260), Swine(26), Others (380).  Total,3043, 99.44%(3043/3060). | Avian (100), Others (11).  Total,111,96.52% (111/115). | Avian (5), Others (1).  Total,6,100% (6/6). | Avian (39).  Total, 39, 100%(39/39). | Avian (92), Human(3),  Others (24).  Total, 119, 100%(119/119). | Avian (10). Total,10,100% (10/10). | Avian (47). Total,47,25% (47/188). | Avian (28). Total,28,100% (28/28). |
| **NS1-226**  **“RGNKMAD”or**  **"RRNKVAD” or**  **“WRNKVAD”or**  **“CRNKVAD”was extended** | Avian (62).  Total,62, 8.00% (62/777). | Avian (17).  Total, 17, 0.56%(17/3060). | Avian (4).  Total,3.48,100% (4/115). | 0 | 0 | 0 | 0 | Avian (118), Others (23). Total,141,75% (141/188). | 0 |
|  |  |  |  |  |  |  |  |  |  |
| **NS2-69 E** | Avian (726), Others (23).  Total,749,100% (749/749). | Avian (2341), Human(261), Others (141).  Total,2743, 99.53% (2743/2756). | Avian (99), Others (11).  Total,110,100% (110/110). | Avian (5), Others (1).  Total,6,100% (6/6). | Avian (39).  Total, 39, 100%(39/39). | Avian (92), Human(3),  Others (24).  Total, 119, 100%(119/119). | Avian (10). Total,10,100% (10/10). | Avian (164), Others (23). Total,187,100% (187/187). | Avian (26). Total,26,100% (26/26). |
| **NS2-69 G** | 0 | Avian (5). Total,5,0.18%(5/2756). | 0 | 0 | 0 | 0 | 0 | 0 | 0 |
| NS2-69 D | 0 | Avian (7). Total,7,0.25%(7/2756). | 0 | 0 | 0 | 0 | 0 | 0 | 0 |
| NS2-69 V | 0 | Avian (1). Total,1,0.03%(7/2756). | 0 | 0 | 0 | 0 | 0 | 0 | 0 |

Note: The protein sequences were obtained from the Influenza Sequences Database ([http://www.ncbi.nlm.nih.gov](http://www.ncbi.nlm.nih.gov/)), up to 18 August, 2016.
